# Evaluation of anti-tumorigenic activity of BP3B against colon cancer with patient-derived tumor xenograft model

**DOI:** 10.1186/s12906-016-1447-8

**Published:** 2016-11-18

**Authors:** Hye-Youn Kim, Jinhee Kim, Huyen Trang Ha Thi, Ok-Sun Bang, Won-Suk Lee, Suntaek Hong

**Affiliations:** 1 Department of Biochemistry, Lee Gil Ya Cancer and Diabetes Institute, Gachon University, 155 Gaetbel-ro Yeonsu-gu, Incheon, 21999 Republic of Korea; 2 KM-Convergence Research Division, Korea Institute of Oriental Medicine, Daejeon, 34054 Republic of Korea; 3 Department of Sugery, Gil Medical Center, Gachon University, Incheon, 21565 Republic of Korea

**Keywords:** BP3B, Colon cancer, Patient-derived tumor xenograft, Medicinal plant

## Abstract

**Background:**

KIOM-CRC#BP3B (BP3B) is a novel herbal prescription that is composed of three plant extracts. Our preliminary study identified that BP3B exhibited potent anti-proliferative activity against various types of cancer cell lines in vitro. Because the in vivo anti-tumor effect of BP3B is not evaluated before clinical trial, we want to test it using patient’s samples.

**Methods:**

To confirm the in vivo anti-cancer effect of BP3B, we used genetically characterized patient-derived colon tumor xenograft (PDTX) mouse model. Anti-cancer activity was evaluated with apoptosis, proliferation, angiogenesis and histological analysis.

**Results:**

Oral administration of BP3B significantly inhibited the tumor growth in two PDTX models. Furthermore, TUNEL assay showed that BP3B induced apoptosis of tumor tissues, which was associated with degradation of PARP and Caspase 8 and activation of Caspase 3. We also observed that BP3B inhibited cancer cell proliferation by down-regulation of Cyclin D1 and induction of p27 proteins. Inhibition of angiogenesis in BP3B-treated group was observed with immunofluorescence staining using CD31 and Tie-2 antibodies.

**Conclusion:**

These findings indicated that BP3B has a strong growth-inhibitory activity against colon cancer in in vivo model and will be a good therapeutic candidate for treatment of refractory colon cancer.

**Electronic supplementary material:**

The online version of this article (doi:10.1186/s12906-016-1447-8) contains supplementary material, which is available to authorized users.

## Background

Colorectal cancer is a serious health problem that has progressively increased to be one of the most common cancers worldwide [1]. Combined therapeutic approaches, such as surgery, chemotherapy and radiation were applied to patients with colorectal cancer. Enormous progress has been made during last decade using fluorouracil (FU) to treat colorectal cancer, with a doubling in dose intensity or tumor response through combined treatment with leucovorin (LV) or bevacizumab or through continuous intravenous administration of FU instead of a bolus injection [2]. In recent years, the chemotherapeutic agents (irinotecan (CPT-11) and oxaliplatin) have resulted in significant progress in the treatment of advanced colorectal cancer. Initial treatment with irinotecan infused FU/LV, commonly known as IFL, resulted in significant increases in response rate, time to disease progression and median overall survival [3, 4]. However, retrospective analyses suggested that the administration of IFL might be limited to patients with a performance status of 0 [5]. When oxaliplatin infused LVFU-2 regimen was compared with a treatment of oxaliplatin plus LVFU-2, known as FOLFOX4, the latter treatment significantly increased the response rate and the time to progression to 9.0 months. The increase in median survival from 14.7 to 16.2 months did not reach statistical significance [6]. Unfortunately, the metastasis and recurrence of colorectal cancer after curative surgery or resistance to chemotherapy eventually lead to a half of colon cancer patients still die [7].

Since cancer is a very complex disease with multiple known and unknown regulatory mechanisms, treating of cancer based on single target mechanism could be less effective than using multi-target strategy [8, 9]. Combination chemotherapies, commonly known as cocktail therapies, targeting diverse abnormalities of cancer, have shown better treatment outcome from various clinical studies [10, 11]. In terms of this fact, natural herbal medicines traditionally used for various disease management, could be valuable sources in developing anti-cancer drugs due to their multi-target/multi-component nature. Herbal prescription may amplify the therapeutic efficacies of each herbal component, exhibiting maximum outcome with less side effects [12]. The traditional usage of these medicinal plants has been successfully performed for preventing asthma, reducing edema, relieving fever, cough, hemorrhages, diarrhea and protecting liver [12].

For research of tumor biology or evaluation of anticancer drugs, in vivo xenograft models have been performed extensively [13, 14]. However, most of these in vivo models are based on a limited number of cancer cells previously isolated from tumors and selected prior to implantation in animals. Unfortunately, these in vivo models were difficult to reproduce the tumor microenvironment and cancer cell adaptation to the innate immune system, both of which are essential to architecture of the primary tumors, proliferation and metastasis [15]. In contrast, patient-derived tumor xenograft (PDTX) model obtained by engraftment of patient biopsies transplanted directly into non-obese diabetic/severe combined immunodeficiency (NOD/SCID) mice, subcutaneously, seems to be able to reduce the biologic differences between the primary patient tumor and the in vivo model [16]. In recent years, PDTX models that have been characterized for predicting drug response in various cancer types [17] and used in numerous preclinical studies [18].

The present study aimed to evaluate the anti-cancer effect of KIOM-CRC#BP3B (BP3B), which is novel herbal prescription of ethanol extract of three medicinal plants on colon cancer using PDTX model. The components of BP3B are *Descurainia sophia* seed, Peucedanum radix (*Peucedanum praeruptorum* Dunn*.*) and *Alnus japonica* branch. Several reports including our previous study identified that the seeds of *D. sophia* contained several cytotoxic and anti-inflammatory substances to induce the death of various cancer cell lines in vitro [19–21]. We also showed that pyranocoumarins from *P. praeruptorum* possessed considerably significant multidrug-resistant reversal activity in multidrug resistant MES-SA/Dx5 cancer cells [22]. *A. japonica* as well contained anti-proliferative and pro-apoptotic compounds that kill some cancer cells such as human leukemia and prostate cancer [23, 24]. Although these three plant materials possessed potential anti-cancer activities, it is very important to measure the anti-cancer activity of herbal mixtures in a reliable in vivo model system. To confirm the anti-tumorigenic activity of BP3B against colon cancer, we investigated the changes of histopathological characteristics, proliferation, angiogenesis and apoptotic cell death in colon tumor tissues.

## Methods

### Reagents

Antibodies against Pecam-1 (M-20:sc-1506),PARP (H-250:sc-7150) and Tie-2 (H-176:sc-9026) were purchased from Santa Cruz Biotechnology (Santa Cruz, CA). Antibodies against p27^kip1^ (#3688) and Cleaved-Caspase-3 (#9661) were obtained from Cell Signaling (Beverly, MA). Anti-Ki67 antibody was purchased from Vector laboratories (Burlingame, CA). Anti-β-actin (A5441) was purchased from Sigma-Aldrich (St. Louis, MO). The 4’6-Diamino-2-phenylindole dihydrochloride was purchased from Thermo Fisher (Waltham, MA).

### Preparation of KIOM-CRC#BP3B

The dried seeds of *Descurainia sophia*, roots of *Peucedanum praeruptorum* Dunn. and branches of *Alnus japonica* were purchased from Kwangmyungdang Medicinal Herbs Co. (Ulsan, Republic of Korea). The identities of each herb material were formally confirmed by Dr. Go Ya Choi, K-Herb Research Center, Korea Institute of Oriental Medicine. All voucher specimens have been deposited at KM-Convergence Research Division, Korea Institute of Oriental Medicine. A whole extract of each herb was separately prepared. In brief, dried plant materials were finely pulverized and immersed in 70% (v/v) ethanol (100 g/L). Then, the solvent extraction was performed by maceration at room temperature (48 h, three times). The extract solutions were filtered through a Whatman filter paper No. 2 (Whatman International, Maidstonem, UK), concentrated using a EYELA rotary evaporation system (Tokyo Rikakikai, Tokyo, Japan) and dried a WiseVen vacuum oven (WOW-70, Daihan Scientific, Seoul, Republic of Korea) to produce a 70% ethanol extract. The dried powder of extract was homogenized and then stored in the dark at 4 °C until use. KIOM-CRC#BP3B was prepared by mixing the three herbal extracts at an equal ratio, 1:1:1 (w/w/w). KIOM-CRC#BP3B was dissolved in 0.5% Na-carboxymethyl cellulose (Na-CMC) solution right before being used in animal experiments.

### Generation of colon PDTX model and in vivo drug efficacy test

The 6-8 week old male *nu/nu* mice (Orient Bio, Seongnam, Korea) were used for in vivo studies and all experiments using immunodeficient mice were carried out in accordance with the guidelines approved by Institutional Animal Care and Use Committees of Gachon University. Fresh surgical tumor tissues (F_0_) were collected immediately after surgery from Gil hospital (Incheon, Korea) and cut into 1 ~ 2 mm^3^-sized pieces in antibiotics-containing RPMI medium. Written informed consent was obtained from each patient and the study was approved by the Gil hospital ethics committee. Tumor fragments were implanted into subcutaneous pockets of mice, which were made in each side of the lower back. When the tumor size reached to 100 ~ 200 mm^3^, those samples were called F_1_, subsequently divided into pieces for passaging in vivo to make F_2_ xenograft tumors. When F2 tumor size reached to 100 ~ 200 mm^3^, collected and cut into 1 ~ 2 mm^3^ sized pieces and implanted into subcutaneous layer on the backs of mice to make F3. When F3 tumor size reached to 100 ~ 200 mm^3^, mice were randomly divided into 4 groups with 5 mice per each group. Mutation status of important cancer-related genes and patient information of colon PDTX samples is listed in Table 1.

**Table 1:** Characteristics of PDTX patient samples

	115 F	102 F
Age	83	50
Sex	F	F
pT (primary status)	3	4a
pM (distant metastasis)	0	1
pN (Lymph node status)	0	2b
Tumor cell type	Adenocarcinoma, Moderately differentiated	Adenocarcinoma, Moderately differentiated
Microsatellite instability	Stable	Stable
KRAS mutation status	G13D	WT
EGFR overexpression	-	-
p53 expression	+	+

One group was treated twice per week with intraperitoneal injection of 5 mg/kg oxaliplatin in PBS and other groups were treated once daily orally with low dose (250 mg/kg) or high dose (500 mg/kg) of BP3B dissolved in 0.5% Na-CMC (w/v). Tumor diameters were serially measured with a digital caliper every 2-3 days and tumor volumes were calculated using the following formula: V = (L × W^2^)/2, V = volume, L = length and W = width. On day 21, mice were sacrificed and tumor tissues were collected, fixed with 10% formalin and embedded in paraffin. Remaining tissues were kept on -80 °C deepfreezer for isolating protein and RNA.

### TUNEL assay

Terminal deoxynuclotidyl transferase-mediated deoxyuridine triphosphate nick-end-labeling (TUNEL) assay was performed to measure nuclear DNA fragmentation in apoptotic cells using DeadEnd™ Fluorometric TUNEL System (Promega, Madison, WI), according to the manufacturer’s instruction. In brief, paraffin sections of colon tumor samples were deparaffinized in xylene and rehydrated in a series of graded alcohols and fixed in 4% paraformaldehyde for 30 min and permeabilized with 20 μg/ml proteinase K for 10 min at room temperature. The tissue section were then incubated with TUNEL reaction buffer in a 37 °C humidified chamber for 1 h, rinsed twice with 2xSSC and PBS and then incubated with DAPI for 1 min at room temperature. Stained apoptotic cells were visualized by fluorescence microscopy.

### Immunohistochemistry and immunofluorescence assay

Paraffin sections of colon tumor samples were deparaffinized in xylene and rehydrated in a series of graded alcohols and antigen was retrieved in 0.01 M sodium citrate buffer. Samples were incubated with 3% H_2_O_2_ for 10 min and followed by 1 h blocking in 1% bovine serum albumin in PBS. The slides were incubated overnight at 4 °C, followed by incubating sections with secondary antibody using ABC kit (Vector laboratories) for 1 h at room temperature. Then, samples were developed with diaminobezidine (Vector Laboratories) reagent and counterstained with hematoxylin and mounted with permount.

To quantify the immunostaining intensity of TUNEL, Ki67, CD31 or Tie-2-positive cells, we used and ImmunoRatio software by analyzing the control and treatment groups. The percentage of positively stained nuclear area was calculated by using a color deconvolution for separating the staining components (diaminobezidine and hematoxylin) in at least 3 fields per each slide. The results were presented as percentage of treated group compared to control one.

### Western blot analysis

Collected colon tumor tissues were lysed in a buffer containing 25 mM HEPES (pH 7.5), 150 mM NaCl, 1% Triton X-100, 10% glycerol, 5 mM EDTA and a protease inhibitor cocktail. Protein concentration was determined using the bicinchoninic acid assay. For western blotting, equal amount of proteins were separated by SDS-polyacrylamide gel electrophoresis, followed by transfer to Immobilon®-P PVDF transfer membrane (Millipore, Bedford, MA). After immunoblotting using specific antibodies, proteins were visualized by chemiluminescence, according to the manufacturer’s instructions (Pierce, Rockford, IL).

### Statistical analysis

These results were represented as the mean ± SD values. The differences between groups were determined using 2-tailed student’s tests and the differences were considered significant when the *P*-values were ≤0.05.

## Results

### BP3B significantly inhibits tumorigenic growth in PDTX model

**Fig. 1:**
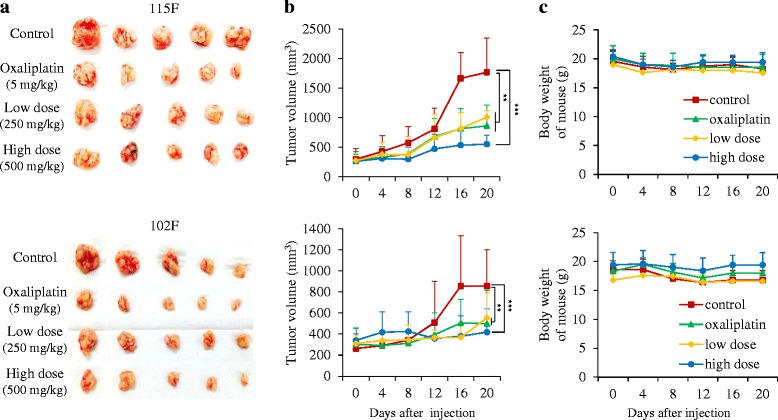
The inhibitory effect of BP3B on in vivo tumor growth in PDTX model. Patient-derived colon tumors were subcutaneously established in BALB/c nude mice. Representative PDTX samples were resected on day 21 (five tumors per group) showing the difference in tumor volumes between vehicle (0.5% Na-CMC), extracts (250 and 500 mg/kg) and oxaliplatin (5 mg/kg) (**a**). When the tumors reached 100-200 mm^3^ in size, mice were treated with drugs for 3 weeks. Tumor sizes were measured every 3 days using a caliper and tumor volumes were calculated (**b**) and body weight (**c**). ** *p* < 0.01 and *** *p* < 0.001

To investigate the ability of BP3B on the inhibition of tumor growth in vivo, *nu/nu* mice were implanted with different types (102 F and 115 F) of patient colon tumors and assigned to the following four groups (n = 5 mice per patient per treatment group) for treatment with 0.5% Na-CMC only (control), oxaliplatin (5 mg/kg), 250 mg/kg or 500 mg/kg of BP3B. As shown in Fig. 1a and b, BP3B administration significantly retarded tumor growth by ~70% compared with control mice. Body weights of the mice were not significantly altered by BP3B administration compared with those of the control group (Fig. 1c) and any defects of other tissues or organs were found in mice. These results suggest that BP3B significantly suppressed tumorigenic growth and did not cause any severe toxicity.

### Histology analysis of xenograft tumors

**Fig. 2:**
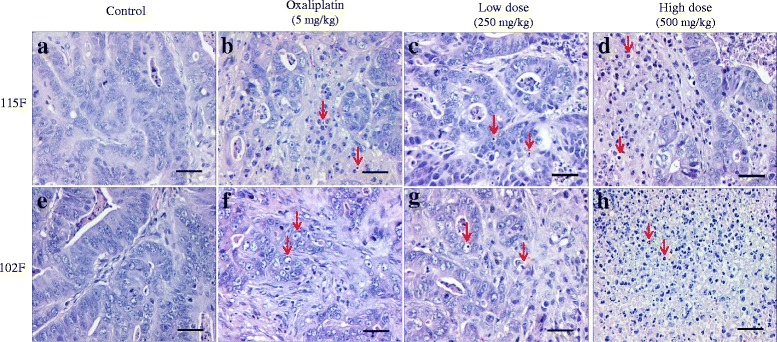
Histological analysis of tumor samples after BP3B administration. After sacrificing the mice, colon tumor tissues from vehicle (**a** and **e**), oxaliplatin (5 mg/kg, **b** and **f**), low dose (250 mg/kg, **c** and **g**) and high dose (500 mg/kg, **d** and **h**)-treated group were fixed and checked with hematoxylin/eosin-staining. Cell nuclei were stained with hematoxylin (*purple*). Red arrows indicate the representative cells with apoptotic characteristics and cell death. Scale bars are 50 μm

To obtain more complete insight into the inhibitory effect of BP3B on tumor growth, histological studies on tumor tissue sections with H&E staining were performed. As shown in Fig. 2, tumor cells in the control group had well-defined cell borders and hyperchromatic nuclei (a and e). However, tumors treated with oxaliplatin (5 mg/kg) (b and f), 250 mg/kg (c and g) or 500 mg/kg (d and h) of BP3B showed significant differences from the corresponding control groups. For instance, tumor cells were accompanied by chromatin condensation, formation of apoptotic bodies. Especially, high dose BP3B-treated tumor tissues revealed more clear apoptotic characteristics and cell death phenotype.

### BP3B treatment promotes apoptotic cell death in xenograft tumors

**Fig. 3:**
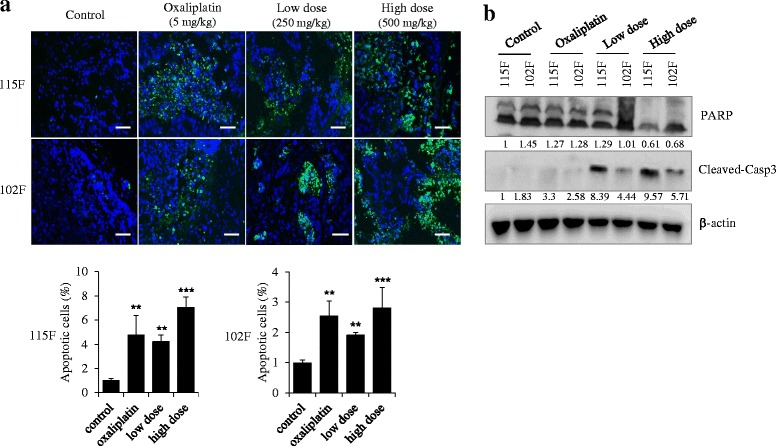
Induction of tumor apoptosis by BP3B in PDTX model. **a** To measure the apoptotic tissues in BP3B-treated tumors, TUNEL assay was performed. Apoptotic cells were visualized into green and nuclei were stained with DAPI (blue). The intensity of image was calculated with ImmunoRatio software. Scale bars are 50 μm. ** *p* < 0.01 and *** *p* < 0.001. **b** Expression pattern of apoptosis-related proteins (PARP and Cleaved-caspase3) was confirmed with western blot after lysis of tumor tissues. Densitometric analysis was performed with Image J software

Based on previous study, we hypothesized that BP3B may have anti-tumor activity through induction of cancer cell apoptosis. Therefore, we assessed whether antitumor activities of BP3B in colon cancer xenografts are exerted through activation of apoptosis. An apoptosis detection kit (TUNEL) was used to identify apoptotic cell death induced by BP3B. As shown in Fig. 3a, oxaliplatin (5 mg/kg), low dose (250 mg/kg) and high dose (500 mg/kg) of BP3B -treated tumor tissues showed significant apoptosis index compared with control group. We also confirmed BP3B mediated cell apoptosis using western blot analysis (Fig. 3b). As a marker for apoptosis, degradation of PARP and activation of Caspase 3 proteins were detected in PDTX samples. As expected, PARP was degraded in drug-treated samples. In opposite way, activated Caspase 3 was increased in two types of PDTX tissues by treatment of BP3B. Although there were no high differences between oxaliplatin and high dose of BP3B-treated 102 F tumors in TUNEL assay, western blot analysis shown the reliable differences of apoptosis between two groups. These data indicate that BP3B induced apoptosis to suppress the tumor growth in PDTX model.

### BP3B regulates cell cycle related proteins in PDTX models

**Fig. 4:**
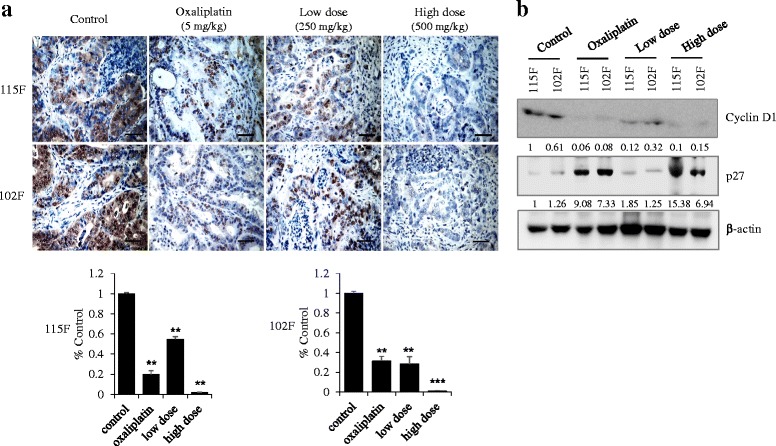
Inhibition of cell proliferation by BP3B in PDTX model. **a** To check the effect of BP3B on cell proliferation, immunohistochemical analysis of Ki67 was performed with drug-treated samples. Ki67 were stained into brown and nuclei were counterstained with hematoxylin (purple). The intensity of Ki67-positive cell was calculated with ImmunoRatio software. Scale bars are 50 μm. * *p* < 0.05, ** *p* < 0.01, *** *p* < 0.001. **b** Expression of cell proliferation markers (Cyclin D1 and p27) was confirmed with western blot. Densitometric analysis was performed with Image J software

To investigate the effect of BP3B on the proliferation of colon cancer cells, we first conducted an immunohistochemistry using intrinsic proliferation marker, Ki67. As shown in Fig. 4a, Ki67 positive cells were highly detected in control groups compared with oxaliplatin and BP3B-treated tumor tissues. This result suggests that BP3B significantly suppresses cell proliferation of in vivo PDTX tumor. To further understand how BP3B inhibits colon cancer cell proliferation, the effect of BP3B on the cell cycle regulators, p27 and cyclin D1, was checked using western blot analysis. As shown in Fig. 4b, cyclin D1 expression was reduced, but p27 expression was increased upon treatment with BP3B. Especially, high dose of BP3B (500 mg/kg) induced significant reduction of cyclin D1 and increase of p27. These results suggest that BP3B inhibits cancer cell proliferation and induces apoptosis by regulating cell cycle related proteins.

### BP3B suppresses angiogenesis in xenograft tumors

**Fig. 5:**
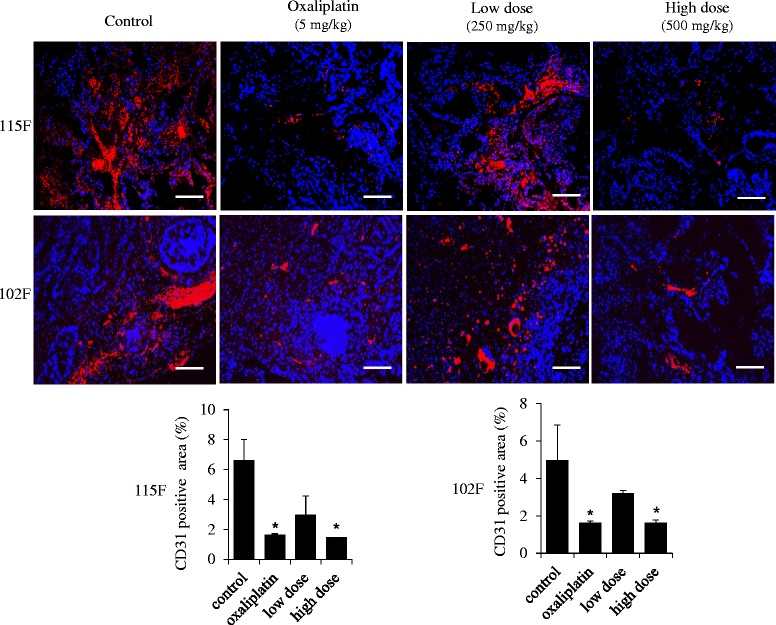
Suppression of angiogenesis by BP3B in PDTX model. To measure the status of tumor angiogenesis in BP3B-treated tumors, immunofluorescence analysis of CD31 was performed. CD31 positive cells were visualized into red and nuclei were stained with DAPI (blue). The intensity of image was quantified with ImmunoRatio software. Scale bars are 50 μm. * *p* < 0.05

Angiogenesis has been known well as an important role in tumor growth, progression and metastasis [25–27] Therefore, we further examined whether BP3B inhibits angiogenesis in xenograft model. Immunofluorescence staining with antibodies against CD31 and Tie-2 revealed that vehicle-treated group formed intact blood vessel in tumor samples. However, oxaliplatin and BP3B-treated groups showed markedly decreased angiogenesis in tumor tissues (Fig. 5 and Additional file 1: Figure S1). These data suggest that BP3B also inhibits the formation of neo-angiogenesis in PDTX tissues.

## Discussion

KIOM-CRC#BP3B is a novel herbal prescription that is composed of three plant materials in equal weighs. *Descurainia sophia* (L.) Weeb ex Prantl (Flixweed) is a member of the family Brassicaceae that is widely distributed in northeastern China and its seed has been broadly used in folk medicine as a cure for throat diseases and viral diseases such as measles and smallpox. Several studies of this plant material have reported that it contains diverse secondary metabolites including cardiac glycoside, flavonoids, lactones, lipids, nor-lignans, and coumarins with various biological activities such as cytotoxicity and anti-inflammatory activity [28, 29]. The dried roots of *Peucedanum praeruptorum* Dunn (Family Umbelliferae) are a well-known traditional Chinese medicine. Several phytochemical and pharmacological studies have shown that various coumarins are the major constituents of this plant and these have diverse biological properties such as anti-inflammatory, chemopreventive and neuroprotective effects [30–32]. *Alnus japonica* is a member of the Family Betulaceae, and the bark of this plant has long been used in traditional medicine in the treatment of fever, hemorrhage, diarrhea, and alcoholism. Several scientific studies revealed that the extract of *A. japonica* contains various biological compounds with anti-adipogenic, anti-proliferative, and anti-parasitic activities [33, 34]. In this study, we showed that BP3B have similar or better therapeutic efficacy compared with oxaliplatin (Fig. 1). Because herbal drugs have been known to exhibit fewer side effects (12), combination therapy with already clinically used drugs, such as oxaliplatin or irrinotecan can increase the anti-cancer activity and show synergistic effect of chemotherapy. Although we did not identify the exact mechanism for anti-tumorigenic activity of BP3B, it will be very valuable information for further study and clinical trial.

Preclinical trial for validation of potential therapeutic targets via in vivo model is regarded as an indispensable procedure for anti-cancer drug development and make the conquest of cancer or other diseases [35]. Recently, many studies have adopted the PDTX technique to perform the preclinical testing of anti-cancer drugs [36]. Previous studies also indicated that transplantation of fresh surgical specimens better represents the cellular and clinical phenotypes of human cancers compared with traditional cell line-based preclinical testing [37, 38]. Histological examination also showed that PDTX model exhibited very similar histology and immunohistochemical phenotypes of patient’s original tumors and maintained the invasive/metastatic features even during serial subtransplantations in vivo. According to their gene expression patterns in breast cancer samples, less than 5% of genes showed variation in expression between PDTX and the homologous primary tumor [37, 39]. Although we did not present in this paper, we also confirmed that the xenograft tumors (F_3_) exhibited similar histologic architecture and pathologic characteristics to that of the original tumors [38]. Established PDTX models of colon cancer were good systems for evaluating the efficacy of anti-tumor drug (Fig. 1). In addition, these models will provide very important tools for developing the novel drugs against drug-resistant tumors, such as cetuximab, using Ras wild-type and mutant samples.

## Conclusion

This study indicated that ethanol extract of dried seeds of *Descurainia sophia*, roots of *Peucedanum praeruptorum* Dunn. and branches of *Alnus japonica* has a strong growth-inhibitory activity against colon cancer in patient-derived tumor xenograft model. BP3B will be a good therapeutic candidate for treatment of refractory colon cancer.

## Additional file

Additional file 1: Figure S1. To measure the status of tumor angiogenesis in BP3B-treated tumors, immunostaining of Tie-2 receptor tyrosine kinase was performed with specific antibody (brown). The intensity of Tie-2-positive cell was quantified with ImmunoRatio software. Cell nuclei were stained with hematoxylin (blue). Scale bars are 50 mm. * p < 0.05. (PDF 421 kb)

## Abbreviations

BP3B: KIOM-CRC#BP3B
FU: Fluorouracil
LV: Leucovorin
PDTX: Patient-derived tumor xenograft
TUNEL: Terminal deoxynuclotidyl transferase-mediated deoxyuridine triphosphate nick-end-labeling.
