# Supplementary material for: Evaluation of anti-tumorigenic activity of BP3B against colon cancer with patient-derived tumor xenograft model
Source: BMC Complement Altern Med. 2016 Nov 18;16:473. doi: 10.1186/s12906-016-1447-8 (PMC5116142; doi:10.1186/s12906-016-1447-8)
Supplement: Additional file 1: Figure S1. — To measure the status of tumor angiogenesis in BP3B-treated tumors, immunostaining of Tie-2 receptor tyrosine kinase was performed with specific antibody (brown). The intensity of Tie-2-positive cell was quantified with ImmunoRatio software. Cell nuclei were stained with hematoxylin (blue). Scale bars are 50 mm. * p < 0.05. (PDF 421 kb) [file 12906_2016_1447_MOESM1_ESM.pdf]

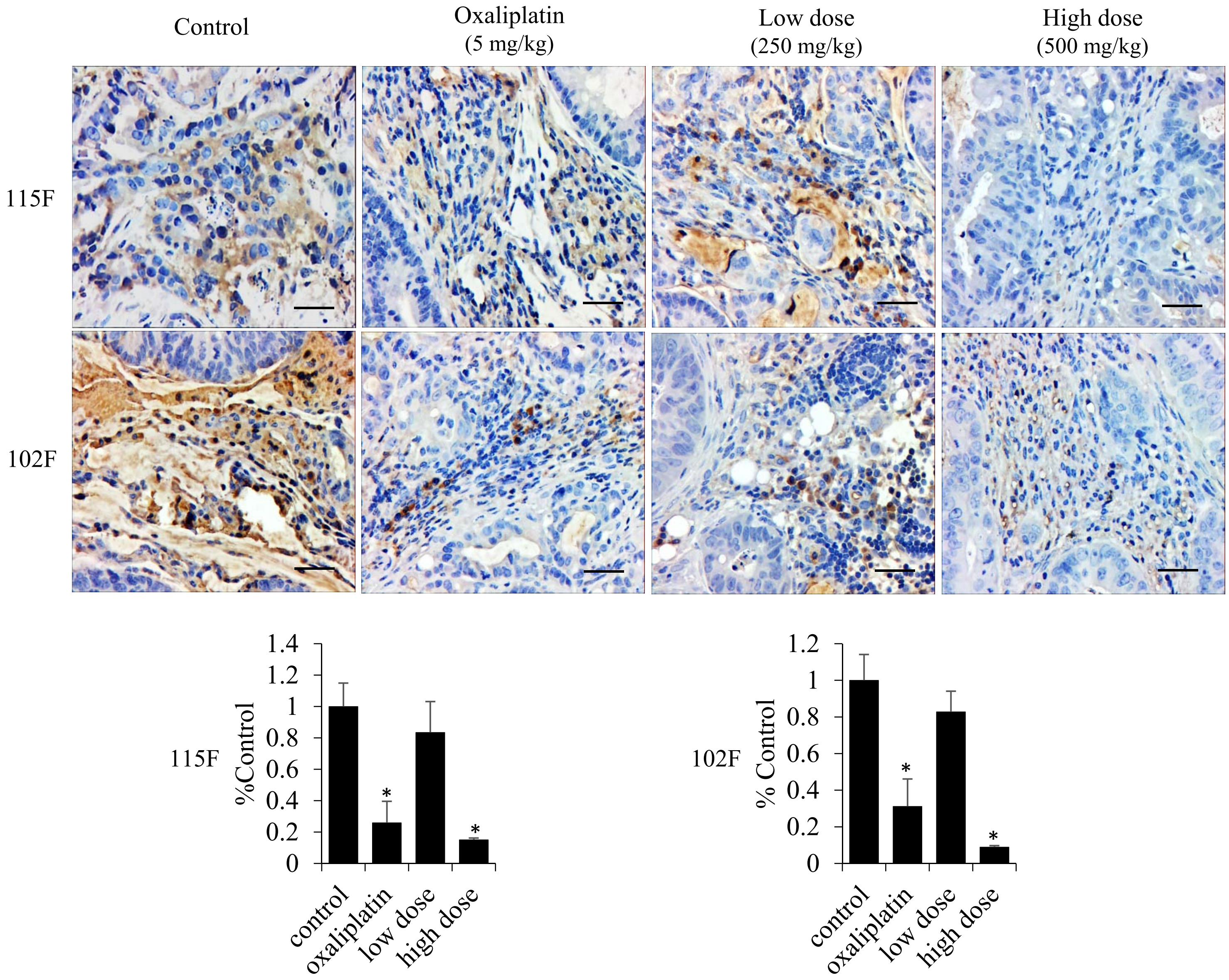

Supplementary Fig. 1 To measure the status of tumor angiogenesis in BP3B-treated tumors, immunostaining of Tie-2 receptor tyrosine kinase was performed with specific antibody (brown). The intensity of Tie-2-positive cell was quantified with ImmunoRatio software. Cell nuclei were stained with hematoxylin (blue). Scale bars are 50 mm. * $p<0.05$.
